# Alzheimer’s Disease as a Membrane Dysfunction Tauopathy? New Insights into the Amyloid Cascade Hypothesis

**DOI:** 10.3390/ijms25179689

**Published:** 2024-09-07

**Authors:** Tomas Olejar, Nikol Jankovska, Radoslav Matej

**Affiliations:** 1 Department of Pathology and Molecular Medicine, Third Faculty of Medicine, Charles University and Thomayer Faculty Hospital, 140 59 Prague, Czech Republic; tomas.olejar@ftn.cz (T.O.); radoslav.matej@ftn.cz (R.M.); 2 Department of Pathology, Third Faculty of Medicine, Charles University and University Hospital Kralovske Vinohrady, 100 34 Prague, Czech Republic; 3 Department of Pathology, First Faculty of Medicine, Charles University and General University Hospital, 128 00 Prague, Czech Republic

**Keywords:** Alzheimer’s disease, tauopathy, amyloid β, membrane dysfunction

## Abstract

The amyloid cascade hypothesis postulates that extracellular deposits of amyloid β (Aβ) are the primary and initial cause leading to the full development of Alzheimer’s disease (AD) with intracellular neurofibrillary tangles; however, the details of this mechanism have not been fully described until now. Our preliminary data, coming from our day-to-day neuropathology practice, show that the primary location of the hyperphosphorylated tau protein is in the vicinity of the cell membrane of dystrophic neurites. This observation inspired us to formulate a hypothesis that presumes an interaction between low-density lipoprotein receptor-related protein 1 (LRP1) and fibrillar aggregates of, particularly, Aβ_42_ anchored at the periphery of neuritic plaques, making internalization of the LRP1-Aβ_42_ complex infeasible and, thus, causing membrane dysfunction, leading to the tauopathy characterized by intracellular accumulation and hyperphosphorylation of the tau protein. Understanding AD as a membrane dysfunction tauopathy may draw attention to new treatment approaches not only targeting Aβ_42_ production but also, perhaps paradoxically, preventing the formation of LRP1-Aβ_42_.

## 1. Introduction

Alzheimer’s disease (AD) is a progressive neurodegenerative disease characterized by the presence of extracellular deposits of β-amyloid peptide (Aβ) in the form of Aβ plaques (also known as senile, neuritic, and amyloid plaques) with different morphologies [1] and an intracellular aggregation of neurofibrillary tangles composed of hyperphosphorylated tau protein [2]. The distribution of tangles in the brain, together with the presence of neuritic plaques (which are a particular subgroup of Aβ plaques distinct from diffuse plaques (i.e., without fibrillar Aβ peptide and dystrophic neurites)), correlates with the clinical development of AD and can be used to characterize the stages of the disease during a neuropathological investigation [3]. Even though the clinical manifestation generally results from neuronal loss and neuronal dysfunction accompanying the accumulation of tau, the mechanism of involvement and, in particular, the “transmembrane” toxicity of Aβ has not been clearly elucidated until now. The amyloid cascade hypothesis (ACH) hypothesizes that extracellular deposits of toxic Aβ are the “initial step” leading to neurodegeneration in AD, which is followed by the accumulation of tau proteins and, subsequently, neuronal death [4]. Verifying or disproving this hypothesis is critical in establishing a causal anti-AD therapy that targets Aβ toxicity [5].

## 2. Preliminary Study: Hyperphosphorylated Protein Tau Aggregates in the Vicinity of the Neuron Membrane

In this work, we present our routine observations showing that hyperphosphorylated tau protein primarily aggregates in the vicinity of the neuronal membrane. This can be seen even using standard methods, which are commonly found in neuropathology practices, including immunofluorescence and confocal microscopy analysis. Pictures showing peripheral tau aggregation in the vicinity of the membrane in a group of patients with an AD–synucleinopathy comorbidity have already been published by our team [6].

**Figure 1:**
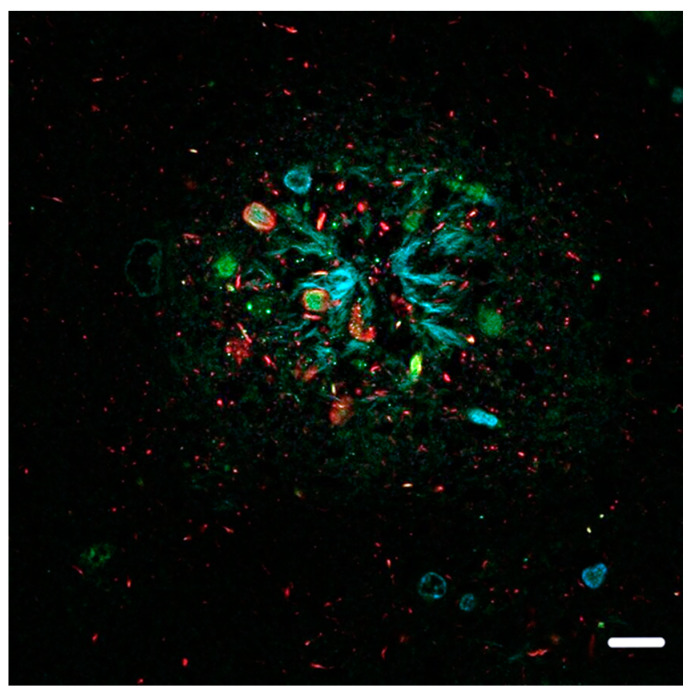
Dilated dystrophic neurites in a neuritic plaque from a subject with comorbid AD–Lewy body dementia (AD-DLB). The hyperphosphorylated tau protein (red), detected using antibody clone AT8, is located mainly in the vicinity of the plasma membrane in dilated dystrophic neurites. The green counterstain shows the accumulation of ubiquitin in the central parts of dystrophic neurites. The blue counterstain shows non-specific DAPI staining of Aβ in Aβ plaques, including contact with the peripheral fibrillar part of dystrophic neurites. The scale bar indicates 10 μm.

**Figure 2:**
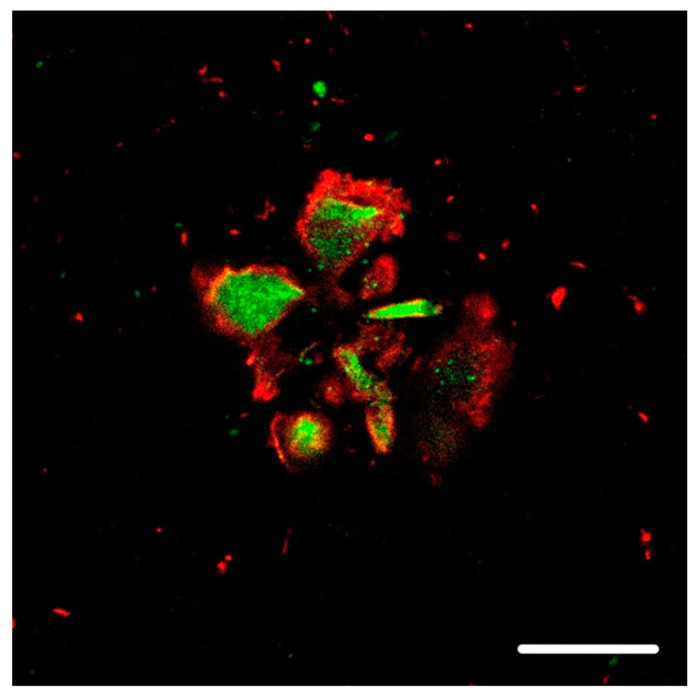
Dilated dystrophic neurites in neuritic plaque from a subject with AD-DLB. The hyperphosphorylated tau protein (red), detected using antibody clone AT8, is located mainly in the vicinity of the plasma membrane in dilated dystrophic neurites. The green counterstain shows the accumulation of ubiquitin in the central parts of dystrophic neurites in neuritic plaque. The scale bar indicates 10 μm.

**Figure 3:**
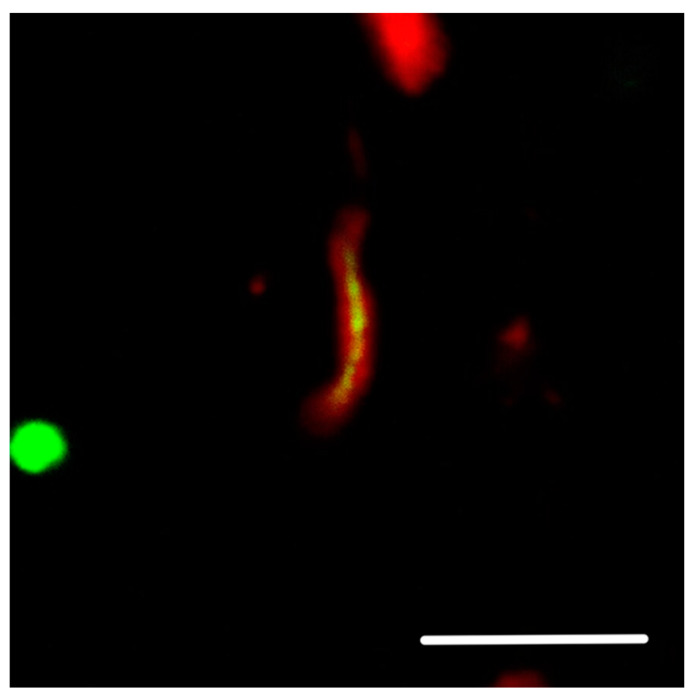
A similar observation can, sometimes—although rarely—also be seen in non-dilated dystrophic neurites (from a subject with AD-DLB). The hyperphosphorylated tau protein (red), detected using antibody clone AT8, is also located mainly in the vicinity of the plasma membrane in non-dilated dystrophic neurites. The green counterstain describes the accumulation of ubiquitin in the central parts of dystrophic neurites. The scale bar indicates 10 μm.

Our neuropathology observations have shown that, in Aβ plaques, hyperphosphorylated tau protein is mainly located in the vicinity of the plasma membrane of dilated, bulbous dystrophic neurites, while the accumulation of ubiquitin is seen in the central parts of these neurites (See Figure 1 and Figure 2). Focusing on this fact and looking at non-dilated neurites, we found membrane-adjacent aggregations of tau protein with a central core composed of ubiquitin (See Figure 3).

## 3. Hypothesis

**Figure 4:**
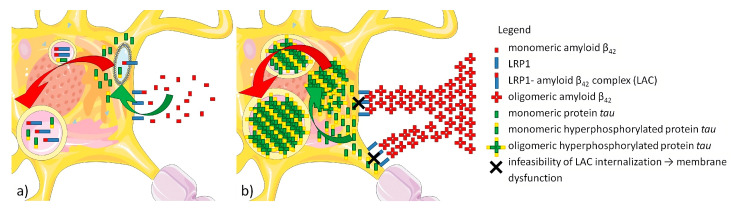
(**a**) Under ideal physiological conditions, non-amyloidogenic or amyloidogenic monomers bind to transmembrane LRP1 receptors, forming a complex that is subsequently internalized and, together with phosphorylated monomers or oligomers of tau, are transported to lysosomes for degradation. (**b**) Under pathological conditions, amyloidogenic oligomers of Aβ_42_ are also able to bind to LRP1 receptors, forming an LRP1-Aβ_42_ complex. However, these oligomers of Aβ_42_ are, parallelly, also linked to the extracellular fibrillar parts located in the periphery of neuritic plaques. This linkage prevents internalization of the LRP1-Aβ_42_ complex and causes severe dysfunction of the cell membrane, either on the uncovered cell membrane of the soma or proximal neurites. Subsequently, membrane dysfunction is accompanied by increased production of tau protein; the hyperphosphorylation of tau protein forms intracellular aggregates that disrupt normal endosomal–lysosomal transport.

We propose that, in AD, low-density lipoprotein receptor-related protein 1 (LRP1) can interact with the fine fibrillar parts of neuritic plaques that are predominantly composed of Aβ_42_. Anchoring Aβ_42_ oligomers in amyloid fibers prevents endocytosis of the LRP1-Aβ_42_ complex and causes serious dysfunction of the neuronal cell membranes and, subsequently, an intracellular response in the form of tau accumulation and hyperphosphorylation in the vicinity of the membrane. Finally, aggregates of hyperphosphorylated tau cause serious dysfunction of ubiquitin–proteasomes, as well as endosomal–lysosomal degradation, which leads to intracellular β-sheet structures and the formation of pathognomonic neurofibrillary tangles. Taken together, we hypothesize that the impotence of the LRP1-Aβ_42_ complex anchored in neuritic plaque to internalize is the causal cell mechanism resulting in neuronal cell membrane dysfunction that leads to the production of hyperphosphorylated tau protein, subsequent damage to intracellular transport pathways, and, finally, to the neuronal death seen in AD (see Figure 4a,b). 

## 4. Supporting Circumstances

(1) As mentioned before, the distribution of neurofibrillary tangles in the brain correlates with the clinical development of AD and characterizes the stages of disease on neuropathological investigation much more than Aβ plaques [7].
(2) Only the number of neuritic plaques, not diffuse plaques, correlate with the severity of AD; only abundant Aβ plaques make the diagnosis definite according to CERAD criteria [8,9].
(3) Aβ plaques, both neuritic or diffuse, are located in the cortex, where neuronal bodies and axonal proximal–initial segments of cortical neurites are not myelinated [10,11]. In these locations, there is enough space either for the production of neuritic plaques or subsequently for inhibiting the internalization of LRP1-Aβ_42_ anchored to Aβ_42_ fibers.
(4) Aβ is not accumulated inside neurons to a significant extent in AD.
(5) According to our long-term observations, hyperphosphorylated tau is primarily located in the vicinity of the plasma membrane; see Figure 1, Figure 2 and Figure 3.

## 5. Animal Models

The 5xFAD murine model using amyloid precursor protein (APP) and Presenilin 1 with five human transgene mutations produces high levels of Aβ, predominantly Aβ_42_ found in Aβ plaques [12,13]. Also, the intracellular location of Aβ was determined in this model, and co-localization with cathepsin D confirmed its presence in lysosomes [14]. Evidence of the intracellular aggregation of tau protein still needs to be clearly demonstrated. The above-mentioned work by Oakley et al. [12] did not report the presence of neurofibrillary tangles, and another work also failed to confirm any neurofibrillary tangles in this model [15]. On the other hand, intracellular thioflavin-positive tau protein aggregates were observed in older 5xFAD animals [16], as well as the hyperphosphorylation of tau [17]. These additional data partly confirm that the ACH indicates the possibility of intracellular tau hyperphosphorylation and aggregation under “pure” Aβ etiopathological conditions. However, this model has a subacute course for AD features, which lasts for months, instead of the chronic course seen in humans that lasts an average of seven decades before the development of clinical and pathological hallmarks of AD. However, the higher intracellular presence of Aβ in the 5xFAD model confirms the key role of receptor-mediated internalization and the intracellular degradation of Aβ oligomers. Contrary to human AD pathology, these high levels of intracellular Aβ and its high-rate metabolic turnover probably cannot result in significant membrane dysfunction and subsequent tau aggregation in the subacute course of the disease seen in the murine transgene model.

Presenilin 1, APP, and tau human transgene mutations are also found in the 3xTg murine AD model where Aβ plaques and neurofibrillary tangles, together with the loss of learning memory, have been reported [18,19]. Unfortunately, tau aggregation in this model is based on the production of an impaired protein that leads to membrane dysfunction different than that hypothesized in this article.

Kanekiyo et al. crossed Lrp1-/- mice with amyloid β-producing mice (APPswe/PSEN1ΔE9) and found increased amounts of amyloid β in the brain tissue [20]. The significant role of neuronal LRP1 in Aβ clearance was confirmed, but, unfortunately, the production of hyperphosphorylated protein tau was not evaluated.

Only an Aβ-producing murine model crossed with Lrp1-/- mice, without a tau protein gene mutation, resulting in the secondary accumulation of tau protein, would be a proper model to evaluate the exact role of LRP1 in the pathology of Alzheimer’s disease.

## 6. Discussion

Aβ is a mix of oligopeptides that are mostly 40–42 amino acids long [21], and, apart from these full-length peptides, the truncated (particularly N-truncated) peptides are the main variants [22,23]. The inner compact parts of neuritic Aβ plaques, having a β-sheet secondary protein structure, are composed predominantly of Aβ_40_ segments [24], which are considered less toxic and less likely to undergo higher-order oligomerization [25,26]. On the other hand, the peripheral fibrillar parts are dominated by the more toxic Aβ_42_ segments [27], which can easily undergo higher-order oligomerization [28,29].

It can be concluded that the simple presence of Aβ plaques by themselves cannot cause the accumulation of tau proteins and the formation of neurofibrillary tangles (which also have the β-sheet secondary structure) that then lead to the above-mentioned neuronal loss and dysfunction [30]. The critical element appears to be a transmembrane mechanism leading to the accumulation of hyperphosphorylated tau in the vicinity of the plasma membrane [31]. The mechanism in question may be linked to the natural Aβ clearance mechanism via the LRP1 receptor, which is a ubiquitous membrane protein that binds different ligands, including Aβ_40_ and Aβ_42,_ or truncated Aβ peptides in the brain, and mediates their endocytosis and degradation [32].

The role of LRP1 in AD is not clearly elucidated [33]. Despite this fact, there are certain reports showing strong LRP1 immunoreactivity in senile plaques and reactive astrocytes in AD subjects, while there is strong immunoreactivity in neurons and light immunoreactivity in astrocytes in patients without dementia [34]. Other authors reported strong LRP1 neuronal positivity, some astrocytic positivity and LRP1-positive senile, particularly cored, plaques in AD brains, as well as in transgenic mice. The control cases without dementia showed only neuronal and astrocytic staining but no LRP1-positive senile plaques [35]. Also, LRP1 mRNA expression was positively correlated with clinicopathological hallmarks of AD, including dementia [36]. It was concluded that the reduced presence of LRP1 in older subjects may increase the probability of AD development [37]. These data may explain the accumulation of extracellular Aβ but not the tau-related pathology. However, tau phosphorylation participates in many processes, including membrane endocytosis [38,39]; thus, neuronal cytoplasmic transport dysfunctions in general are accompanied by increased tau phosphorylation that may subsequently facilitate its aggregation [40].

## 7. Conclusions

This hypothesis, as presented, arises from the classic ACH formulated by Hardy and Higgins in 1992, which determined that extracellular aggregation of Aβ is the initial mechanism in the development of AD neuropathology [4]. In our understanding, breaking the physiological internalization of the Aβ_42_-LRP1 complexes with Aβ_42_ anchored in amyloid fibrils causes a gradual dysfunction of unmyelinated neuronal membranes, resulting in classic “tauopathy.” This theory anticipates two substantial considerations: (1) other “tauopathies” are probably caused by either extracellular or intracellular membrane dysfunction, and (2) simply targeting Aβ_42_ fibrils will not lead to an effective anti-AD therapy. Paradoxically, the early blocking of LRP1-Aβ_42_ complexes accompanied by enhanced brain “flushing” may be a reasonable method.

## 8. Material and Methods

### 8.1. Patients

We present observations of two randomly selected female patients, aged 86 and 88, from our neuropathology practice, with AD comorbid with synucleinopathy.

### 8.2. Tissue Samples

Brain tissue samples were fixed for 3–4 weeks in buffered 10% formalin. Then, using the BrainNet Europe standardized protocol [41,42], selected tissue blocks were embedded in paraffin using an automatic tissue processor. Sections (5 μm thick) were prepared and stained with hematoxylin-eosin, Klüver–Barrera, and silver impregnation methods. For analysis, representative blocks of the left hippocampal and parahippocampal areas were chosen.

### 8.3. Immunofluorescence and Immunohistochemistry

Briefly, 5 μm thick sections of formalin-fixed and paraffin-embedded tissue samples were deparaffinized and then incubated with primary antibodies for 20 min at room temperature. For confocal microscopy, secondary antibodies conjugated to Alexa Fluor^®^ (Thermo Fischer Scientific, Waltham, MA, USA, see below) were used. Paraffin sections were also treated with 20× TrueBlack^®^ (Biotium 23007, Fremont, CA, USA) diluted in 1× 70% alcohol to quench lipofuscin autofluorescence.

#### 8.3.1. Primary Antibodies

For immunohistochemistry, 5 µm thick sections of formalin-fixed and paraffin-embedded tissue were selected from the left hippocampal region, including the entorhinal and transentorhinal cortex. These were incubated with primary antibodies against the following antigens: (1) Phospho-Tau (Ser202, Thr205) Monoclonal Antibody (1:500, mouse monoclonal, clone AT8; Thermo Fisher Scientific MN1020, Waltham, MA, USA), and (2) Ubiquitin (1:2000, mouse monoclonal, clone Ubi-1; MilliporeSigma MAB1510-I-25UG, Burlington, MA, USA).

#### 8.3.2. Secondary Antibodies

For the detection of immunostaining, we used horseradish peroxidase–diaminobenzidine (see above) for immunohistochemistry and secondary antibodies conjugated with Alexa Fluor^®^ 488 (1:1000, donkey anti-rabbit, H + L IgG, Thermo Fischer Scientific, Waltham, MA, USA), and Alexa Fluor^®^ 568 (1:1000, donkey anti-mouse, H + L IgG, Thermo Fischer Scientific, Waltham, MA, USA) was used for immunofluorescence staining. Slides incubated with only the secondary antibody were used as specificity controls. VECTASHIELD^®^ Mounting Medium with DAPI (Vector Laboratories Ltd., London, UK) was used as a nonspecific stain for Aβ [43] and as a nuclear counterstain.

### 8.4. Confocal Microscopy

#### 8.4.1. Figure 1

The colocalization of protein aggregates was imaged using a Leica TCS SP5 confocal fluorescent laser scanning microscope (Leica Microsystems Inc., Wetzlar, Germany). An HCX PL APO objective was used with 40× magnification, oil immersion, and a 1 AU pinhole. Donkey anti-Rabbit IgG secondary antibody was conjugated to Alexa Fluor^®^ 488 and excited at 488 nm using a 65 mW multi-line argon laser, whereas donkey anti-mouse IgG conjugated to Alexa Fluor^®^ 568 was excited at 561 nm using a 20 mW DPSS laser.

#### 8.4.2. Figure 2 and Figure 3

The colocalization of protein aggregates was imaged using a Leica STELLARIS 8 FALCON confocal fluorescent laser scanning microscope (Leica Microsystems Inc., Wetzlar, Germany). An HC PL APO 63×/1.40 OIL CS2; FWD 0.14 MM| BF, POL, DIC objective was used with oil immersion. Donkey anti-rabbit IgG secondary antibody was conjugated to Alexa Fluor^®^ 488, and donkey anti-mouse IgG conjugated to Alexa Fluor^®^ 568 was excited using 440–790 nm pulsed white light laser with a pulse picker (WLL PP).

## Data Availability

The authors confirm that all data underlying the findings are fully available without restriction. All data are included in the manuscript.
